# Splenic sympathetic signaling contributes to acute neutrophil infiltration of the injured spinal cord

**DOI:** 10.1186/s12974-020-01945-8

**Published:** 2020-09-23

**Authors:** Susana Monteiro, Andreia G. Pinho, Mara Macieira, Cláudia Serre-Miranda, Jorge R. Cibrão, Rui Lima, Carina Soares-Cunha, Natália L. Vasconcelos, José Lentilhas-Graça, Sara Duarte-Silva, Alice Miranda, Margarida Correia-Neves, António J. Salgado, Nuno A. Silva

**Affiliations:** 1 Life and Health Sciences Research Institute (ICVS), School of Medicine, University of Minho, Campus de Gualtar, 4710-057 Braga, Portugal; 2 ICVS/3B’s—PT Government Associate Laboratory, Braga, Portugal

**Keywords:** Spinal cord injury, Spleen, Sympathetic fibers, Neutrophils

## Abstract

**Background:**

Alterations in the immune system are a complication of spinal cord injury (SCI) and have been linked to an excessive sympathetic outflow to lymphoid organs. Still unknown is whether these peripheral immune changes also contribute for the deleterious inflammatory response mounted at the injured spinal cord.

**Methods:**

We analyzed different molecular outputs of the splenic sympathetic signaling for the first 24 h after a thoracic compression SCI. We also analyzed the effect of ablating the splenic sympathetic signaling to the innate immune and inflammatory response at the spleen and spinal cord 24 h after injury.

**Results:**

We found that norepinephrine (NE) levels were already raised at this time-point. Low doses of NE stimulation of splenocytes in vitro mainly affected the neutrophils’ population promoting an increase in both frequency and numbers. Interestingly, the interruption of the sympathetic communication to the spleen, by ablating the splenic nerve, resulted in reduced frequencies and numbers of neutrophils both at the spleen and spinal cord 1 day post-injury.

**Conclusion:**

Collectively, our data demonstrates that the splenic sympathetic signaling is involved in the infiltration of neutrophils after spinal cord injury. Our findings give new mechanistic insights into the dysfunctional regulation of the inflammatory response mounted at the injured spinal cord.

## Background

Spinal cord injury (SCI), especially when affecting upper anatomical levels, can impair the immune system function leading to increased susceptibility for infections [1–5]. Trauma to the cervical or thoracic part of the spinal cord damages supraspinal autonomic projections and sympathetic preganglionic neurons located at the intermediolateral nucleus (IML). These are crucial for relaying autonomic information from the central nervous system to the peripheral sympathetic postganglionic neurons innervating lymphoid organs. The sympathetic signaling can become further compromised by the increased intraspinal plasticity following the primary injury that through the re-organization of sympathetic pathways results in erroneous reflexive circuits [4, 5]. This maladaptive plasticity has been shown to underlie autonomic dysreflexia causing immunosuppression [4, 5] through alterations in the sympathetic communication to the spleen.

The spleen is a privileged site for neuroimmune communication. The splenic parenchyma is rich in sympathetic innervations expressing both synaptophysin and tyrosine hydroxylase, displaying a uniquely complex and panicle-like neuronal architecture [6]. The morphology of the splenic neuronal arborization contrasts with that of other lymphoid organs such as the lymph nodes or the thymus where it is mostly abundant at the organ surfaces [6]. The splenic sympathetic signaling is crucial for proper immune function and is linked to important steps of inflammation such as immune cell recruitment and mobilization [7, 8], and innate response to pathogens [6] and to extramedullary hematopoiesis [9, 10].

The spleen is central for erythrocyte recycling and immune response to pathogens. Nevertheless, it also discloses an important role in wound healing. Indeed, immune cells in the spleen can become rapidly activated and mobilized to sites of inflammation and tissue injury. One example are monocytes residing in the subcapsular zone of the red pulp that in response to myocardial ischemia are rapidly mobilized to the inflamed tissue participating in the repair process [11]. Moreover, it was demonstrated that the splenic nerve activity was associated with T cell egress from the spleen contributing for hypertensive pathology. Conversely, the ablation of the splenic nerve prevented not only the egress of splenic T lymphocytes and their infiltration into target organs as also prevented hypertensive episodes [12], indicating splenic nerve signaling as a regulator of the immune response also in pathological context. SCI can severely impact spleen morphology and function although most alterations are documented at the chronic phase. Some of these include increased levels of norepinephrine (NE) [2, 13], hyper sympathetic innervation [14], atrophy, and changes in immune cell frequencies [1, 3, 4]. The deregulation of the systemic immune response has been associated with overactive sympathetic signaling to lymphoid organs and to excessive activation of the hypothalamic-pituitary-adrenals axis (HPA).

Interestingly, many aspects of the intraspinal inflammatory response after SCI are also examples of a non-regulated immune response. Both local immune players—microglia and peripheral infiltrative cells—progressively display highly inflammatory phenotypes contributing for extending the initial damage and not participating in wound healing. Moreover, these cells persist for long periods of time in the spinal cord tissue and become part of the secondary injury that characterizes the chronic phase of SCI pathophysiology. The dysfunctional and non-resolving features of SCI-associated intraspinal inflammation have mainly been attributed to local environmental cues, and the involvement of a systemic deregulation of the immune response is mostly overlooked. Some lines of evidence demonstrate that the peripheral infiltration of myeloid cells affects local microglia responses diminishing phagocytic capacity and altering microglial cell death [15], highlighting the importance of peripheral inflammatory cells in shaping local responses. In fact, the first monocyte-derived macrophages present in the spinal cord after injury are originated from the spleen, and importantly, removal of the spleen improves spontaneous motor recovery of SCI animals [12, 16–18], suggesting a detrimental role for these peripheral cells.

Here, we questioned whether SCI-induced alterations in the sympathetic signaling of the spleen, conveyed by the splenic nerve, could be affecting the early presence of peripheral inflammatory cells in the spinal cord. At first, we show that within the first 24 h after SCI increased levels of NE were the most significantly altered correlate of sympathetic function at the spleen. Importantly, in vitro data demonstrated that NE stimulation of splenic myeloid populations affects preferentially neutrophils that respond by increasing their numbers. In line with the in vitro data, ablation of the sympathetic activation through splenic sympathetic denervation resulted in less neutrophils in the spleen 24 h after SCI. Finally, we demonstrated that the ablation of the splenic nerve reduces the number of neutrophils in the spinal cord after the injury, implying that the sympathetic signaling to the spleen is involved in the acute infiltration of inflammatory neutrophils after injury.

## Methods

### Animals

The Ethical Subcommittee in Life and Health Sciences (SECVS; ID: 018/2019, University of Minho) previously approved all experiments. Local regulations on animal care and experimentation (European Union Directive 2010/63/EU) were respected. Female C57BL/6 J mice (Charles River, USA), 10–15 weeks, were maintained at the animal facilities of the Institute of Life and Health Sciences (ICVS, Braga, Portugal) under sterile conditions and in light, humidity, and temperature-controlled rooms. Animals had food and water provided ad libitum.

### Spinal cord injury surgery and post-operative care

All experiments were approved by the Ethical Subcommittee in Life and Health Sciences (SECVS; ID: 018/2019, University of Minho) and conducted in accordance with the local regulations on animal care and experimentation (European Union Directive 2010/63/EU). C57BL/6 J mouse (Charles River, USA) was maintained under standard laboratory conditions (12 h light: 12 h dark cycles, 22 °C, relative humidity of 55%, ad libitum access to standard food and water). C57BL/6 J adult females (10–15 weeks age) were used for the in vivo study. Animals were handled every other day for 1 week prior to the surgeries, for stress reduction and habituation to the experimenter presence. Animals were anesthetized with an intraperitoneal injection of a mixture containing ketamine (Imalgene, 75 mg/kg, Merial, France) and medetomidine (Dormitor, 1 mg/kg, Pfizer, USA). Once anesthetized, buprenorphine (Bupaq, 0.05 mg/kg) was also administrated subcutaneously for analgesia. To confirm the surgical plane of anesthesia, mice were pinched on the lower limb toes. Mice were placed under a warm lamp before and after surgery and placed on a warm surgical blanket during surgery to avoid dropping of the body temperature. Vaseline was applied onto the eyes to prevent drying and corneal damage. Surgeries were performed following aseptic procedures. A dorsal midline incision was made at the level of the thoracic spine (T5-T12). The paravertebral muscles were retracted and the spinous and laminar arc of T8-T9 removed, exposing the spinal cord. Using forceps (Dumont #5/45° angled, FST, CA, USA), the spinal cord was completely compressed for 10 s to produce a severe lesion. Lesions were performed always with the same forceps and always by the same experimenter to reduce variability. Immediately after the surgical procedure, the skin was closed with 9-mm autoclips (Braintree Scientific, USA). Atipamezole (Antisedan/Pfizer) was administered subcutaneously to revert the anesthesia, and animals were allowed to completely recover from the anesthesia under a warm lamp. Animals were then placed on individual cages with hydrogel and moisturized food pellet on the floor to allow an easy access to water and food. Post-operative care included buprenorphine (Bupaq, 0.05 mg/kg), vitamins (Duphalyte, Pfizer, New York, NY, USA), saline, and enrofloxacin (Bayer, Leverkusen, Germany) administration, twice a day. Manual expression of bladders was performed twice a day. During post-operative care, animals were also monitored for body temperature and recovery of general activities. For the 24 hpi analysis, the Basso Mouse Scale (BMS) [19] was used prior to animals’ occision to confirm the lesion extent reflected by no motor movement in both hind paws (BMS score = 0). Animals were placed in an open arena, and their locomotor function was scored according to the BMS scale by two independent observers, blinded to the experimental groups. BMS scoring was performed as a quality control procedure for the surgery to exclude eventual partial lesions.

### Splenic denervation surgery

All animals received an intraperitoneal injection with anesthetic, a mixture of ketamine (Ketamidor, Richeter Pharma AG, Austria) and medetomidine (Dormitor, 1 mg/kg, Pfizer, USA). Once anesthetized, buprenorphine (Bupaq, 0.05 mg/kg) was also administrated subcutaneously for analgesia. Vaseline was applied onto the eyes to prevent drying and corneal damage. A heat lamp and a surgical blanket kept the body temperature from falling. After an abdominal midline incision, the stomach was retracted upwards, and the spleen was stabilized using retractors. The splenic artery was exposed. A thermal cautery was carefully placed near the splenic artery, for 2–3 s until it dilated, resulting in thermoablation of the splenic nerve [12]. The abdominal cavity was bathed with warmed saline 0.9%. Sham animals had the same surgery, except for the thermoablation step. Abdominal muscles and skin were sutured at the end of the surgical procedure. Atipamezole (Antisedan (Orion Corporation, Finland)) was administered subcutaneously to revert the anesthetic state.

### Splenectomy surgery

All animals received an intraperitoneal injection with anesthetic, a mixture of ketamine (Ketamidor, Richeter Pharma AG, Austria) and medetomidine (Dormitor, 1 mg/kg, Pfizer, USA). Once anesthetized, buprenorphine (Bupaq, 0.05 mg/kg) was also administrated subcutaneously for analgesia. Vaseline was applied onto the eyes to prevent drying and corneal damage. A heat lamp and a surgical blanket kept the body temperature from falling. After lateral left flank incision, the spleen was exposed and removed with forceps and scissors. The abdominal cavity was bathed with warmed saline 0.9%. Sham animals had the spleen exposed but was not removed. Abdominal muscles and skin were sutured at the end of the surgical procedure. SCI (or laminectomy only) was applied after the splenectomy. Atipamezole (Antisedan (Orion Corporation, Finland)) was administered subcutaneously to revert the anesthetic state.

### Histological assessment

Mice were anesthetized and transcardially perfused with 20 ml of cold phosphate-buffered saline (PBS) and then with 4% paraformaldehyde/PBS (PFA). Spleen was dissected and post-fixed for 24 h in 4% PFA. The spleen was then cryoprotected in a 30% sucrose solution for 24 h and involved in optimal cutting temperature compound (OCT), Thermo Scientific, snap-frozen in liquid nitrogen, and stored at − 20 °C. Spleens were sectioned in a cryostat (Leica CM1900, LeicaBiosystems, Nussloch, Germany), and 20 μm tissue sections were mounted onto charged slides. All histological procedures and evaluation were performed blindly to the treatment groups.

### Hematoxylin-eosin staining

Tissue sections were stained for hematoxylin-eosin to assess splenic histoarchitecture. Microphotographs were collected using bright field light microscopy (Olympus Widefield Upright Microscope BX61, Tokyo, Japan, equipped with an Olympus DP-70 digital camera), using a × 20 magnification objective and processed by the *cellSens* software. Using the Multi-Image Alignment (MIA) algorithm, the whole section was scanned and reconstructed.

### Splenocytes in vitro stimulation

Mice were anesthetized and transcardially perfused with 20 ml of cold PBS. The spleen was isolated and kept in ice-cold DMEM. Single-cell suspensions of the spleen and spinal cord were made by mechanical dissociation in Dulbecco’s modified eagle medium (DMEM) with 10% heat-inactivated fetal calf serum (FCS) and 1% penicillin-streptomycin. Erythrocytes were depleted with ammonium-chloride-potassium (ACK) lysis solution. The cell pellet was then washed, re-suspended in DMEM, and manually counted using a hemocytometer. 1 × 10^6^ cells of splenocytes were plated and stimulated with NE (Sigma-Aldrich, Saint Louis, CA, USA) different concentrations for 6 h in an incubator at 37%, 5% CO_2_. After washing in fluorescence-activated cell sorting (FACS) buffer (PBS, 10% bovine serum albumin (BSA), 0.1% azide) to remove NE, cells were re-counted and followed the staining protocol for flow cytometry. Briefly, the Fc portion was blocked using anti-mouse CD16/CD32 (Biolegend). Then surface cell staining was performed by incubating a cocktail of antibodies for 30 min at 4 °C in. After washing, cells were re-suspended in 200 μl of FACS buffer. Precision counting beads (Biolegend) were added to the single cell suspensions according to manufacturer instructions to calculate final cell concentrations, acquired in a LSRII Flow Cytometer (BD, Pharminogen, California, USA) and analyzed with the Flow Jo software version 10.4.

### Immunohistochemistry protocol

For immunofluorescence staining, sections of the spleen and the whole splenic arteries were washed in PBS, permeabilized with 0.2% Triton X-100 for 10 min, and blocked with 2% FCS in 0.2% Triton X-100 for 30 min. Afterwards, the primary antibody was incubated overnight at room temperature: rabbit tyrosine hydroxylase (TH) (1:500). In the following day, sections were incubated with the secondary antibody goat anti-rabbit Alexa 594 (1:1000; Invitrogen, Paisley UK). Sections were counterstained with 4′,6-diamidino-2-phenylindole (DAPI) for 30 min (1:1000; Sigma-Aldrich, Saint Louis, CA, USA) and mounted with Immu-Mount® (Thermo Scientific, Waltham, MA, USA). Between steps, 5 washes with PBS (1×) were performed. Spleen sections were scanned for TH+ staining in fiber-like structures, and non-overlapping images were acquired using the Olympus Widefield Inverted Microscope IX81, Tokyo, Japan. Fiber density analysis was performed by measuring the percentage of section area occupied by TH+ fiber using the *ImageJ v1.52* software. Whole splenic artery images were acquired using confocal point-scanning microscope, Olympus FV1000. All histological procedures and evaluation were performed blindly to the treatment groups. A total of 6 spleen sections per animal were analyzed, and TH positivity was normalized to total section area.

### Flow cytometry of the spleen and spinal cord tissue

Mice were anesthetized and transcardially perfused with 20 ml of cold PBS. The spleen was isolated and kept in ice-cold DMEM. The spinal cord was rapidly isolated from the vertebral column and 1 cm of the spinal cord (centered at the epicenter of the lesion) kept in ice-cold DMEM. Single-cell suspensions of the spleen and spinal cord were made by mechanical dissociation in DMEM with 10% heat-inactivated FCS and 1% penicillin-streptomycin. Erythrocytes were depleted with ACK lysis solution. Spinal cord cell suspensions were passed through a 70-μm mesh. Myelin debris was removed through centrifugation in a 37% Percoll gradient. The cell pellet was then washed in FACS buffer (PBS, 10% BSA, 0.1% azide). 1 × 10^6^ cells of spleen cells were stained. The Fc portion was blocked using anti-mouse CD16/CD32 (Biolegend). Then surface cell staining was performed by incubating a cocktail of antibodies for 30 min at 4 °C in. After washing, cells were re-suspended in 200 μl of FACS buffer. Precision counting beads (Biolegend) were added to the single cell suspensions according to manufacturer instructions to calculate final cell concentrations. Cells and beads were acquired in a LSRII Flow Cytometer (BD, Pharminogen, California, USA) and analyzed with the *Flow Jo* software version 10.4. The gating strategy used can be found in supplementary data.

### High-performance liquid chromatography (HPLC) quantification of catecholamines

The spleen was collected 1, 6, and 24 h after SCI, the tissue was divided in two, one half was used for HPLC, and another half was used for real-time polymerase chain reaction (qPCR), both samples were rapidly snap-frozen in liquid nitrogen and stored at − 80 °C. The spleens were weighed, and then, catecholamines were extracted after homogenization in 200 μL of perchloric acid. Samples were sonicated and then centrifuged at 10,000 rotations per minute (RPM) for 10 min. Resulting supernatants were filtered through a Spin-X HPLC column (Costar) to remove debris. Levels of dopamine, NE, epinephrine, and serotonin were measured by HPLC combined with electrochemical detection using a Gilson instrument, fitted with an analytical column (Supelco Supelcosil LC-18 3 mM, flow rate: 10 ml/min) as previously described [20]. Briefly, 150 ml supernatant aliquots were injected into the system, using a mobile phase of 0.7 M aqueous potassium phosphate (pH 3.0) in 10% methanol, 1-heptanesulfonic acid (222 mg/l), and Na-EDTA (40 mg/l). A standard curve using known concentrations of NE, epinephrine, and dopamine was run.

### Real-time quantitative polymerase chain reaction (qPCR) for measuring gene expression

Stored frozen spleen tissue (− 80 °C) was homogenized in TripleXtractor (Grisp, Porto, Portugal), and total ribonucleic acid (RNA) was extracted as directed by manufacturer’s instructions and dissolved in ultrapure RNAse-free water. Synthesis of complementary deoxyribonucleic acid (cDNA) was performed using Xpert cDNA Synthesis Mastermix reverse transcriptase kit (Grisp, Porto, Portugal) from 1.5 μg of total RNA template. mRNA levels were analyzed by real-time qPCR using the Xpert Fast SYBR Mastermix (Grisp, Porto, Portugal) according to the manufacturer’s instructions and using the oligonucleotides primers. qPCR reactions were performed in a Bio-Rad CFX 96 real-time PCR detection system (Bio-Rad, California, USA). Melting curve analysis was used to assess the specificity of the gene amplification. Gene expression was normalized to three housekeeping genes: glyceraldehyde 3-phosphate dehydrogenase (*Gadph*), hypoxanthine-guanine phosphoribosyltransferase (*Hprt*), and *18S* gene expression.

### Oligonucleotide primers

**Table:** 

Gene	Forward	Reverse
***18 s***	5-GTA ACC CGT TGA ACC CCA TT-3	5-CCA TCC AAT CGG TAG TAG CG -3
***Bdnf***	5-GCG CCC ATG AAA GAA GTA AA-3	5-TTC GAT GAC GTG CTC AAA AG-3
***Gadph***	5-GGG CCC ACT TGA AGG GTG GA-3	5-TGG ACT GTG GTC ATG AGC CCT T-3
***Gap43***	5-ATA ACT CCC CGT CCT CCA AGG C-3	5-ACA GCG TCT TTC TCC TCC TCA GC-3
***Hprt***	5-GCT GGT GAA AAG GAC CTC T-3	5-CAC AGG ACT AGA ACA CCT GC-3
***MaoA***	5-ATG AGG TAT CTG CCC TGT GG-3	5-CTC TCA GGT GGA AGC TCT GG-3
***Net***	5-GTG AAG GAA CGC AAC GGA GT-3	5-CAG GAA GGC ACC ACC ACC AT-3
***Ngf***	5-GAG GGG AGC GCA TCG AGT T-3	5-GGT TCT GCC TGT ACG CCG AT-3
***Sema3A***	5-TCC ACG GCC AGG AAC TTG TC-3	5-GCC ATC TTC TGC ATC CAC TCG-3

### Western blot analysis

The spleen was collected 1 and 15 dpi, weighted and rapidly snap-frozen in liquid nitrogen, and stored at − 80 °C. Half-spleen was homogenized in ice-cold RIPA buffer with protease inhibitor cocktail (cOmplete; Roche, Switzerland). Samples were sonicated for 5 min, centrifuged, and supernatant collected for protein quantification. Bradford assay (BioRad, USA) was used for protein quantification. Samples were boiled at 100 °C for 5 min with Laemli buffer and β-mercaptoethanol. Twenty micrograms of total protein was loaded into a 15% SDS-Page gel and then transferred to a nitrocellulose membrane. After overnight incubation at 4 °C with primary antibodies: rabbit anti-TH (1:1000, Millipore, USA), mouse anti-βIII tubulin (1:1000, Promega, UK), mouse anti-βactin (1:1000, Abcam, UK), the secondary antibodies were incubated for 1 h at room temperature following dilutions: anti-rabbit-HRP (1:10.000, BioRad, USA) and anti-mouse-HRP (1:10.000, BioRad, USA). Antibody binding was assessed by chemiluminescence (ECL kit, BioRad, USA) in a Sapphire Biomolecular Imager (Azure Biosystems, USA). Band quantification was performed using the Azure Spot Analysis software (Azure Biosystems, USA) according to manufacturer’s instructions using β-actin as the loading control.

### Antibody list

**Table:** 

Marker	Fluo.	Company	Target	
7-AAD	PerCpCy5.5	Biolegend	Dead cells	1/80
CD11b	PE	Biolegend	Myeloid cells	1/200
CD11c	BV 605	Biolegend	Mostly dendritic cells	1/100
NK 1.1	BV 510	Biolegend	Natural killer	1/200
CD19	FITC	Biolegend	B lymphocytes	1/200
CD3	APC	Biolegend	T lymphocytes	1/100
CD45	PeCy7	Biolegend	Leukocytes	1/200
Ly6C	BV711	Biolegend	Monocytes *	1/100
Ly6G	BV650	Biolegend	Granulocytes	1/100
Purified anti CD16/32	None	Biolegend	Fc Block	1/25
TH	None	Millipore	Sympathetic fibers	1/500

### Statistical analysis

Statistical analysis was performed using *GraphPadPrism* ver.6.0. Independent sample *t* test or Mann-Whitney was used when comparing two groups. When comparing three groups, one-way ANOVA, followed by Tukey test was used or the Kruskal-Wallis test was used, followed by Dunn’s multiple comparisons test. Two-way ANOVA was used when comparing two factors followed by Sidak’s multiple comparisons test. Normality was measured using the Kolmogorov-Smirnov and Shapiro-Wilk statistical tests. Equality of variances was measured using the Levene’s test and was assumed when *p* > .05. Values were accepted as significant if the *p* < .05. Data presented as group mean ± standard error of the mean (SEM).

## Results

### Mid-thoracic SCI triggers a progressive remodeling of the splenic sympathetic innervation

**Fig. 1:**
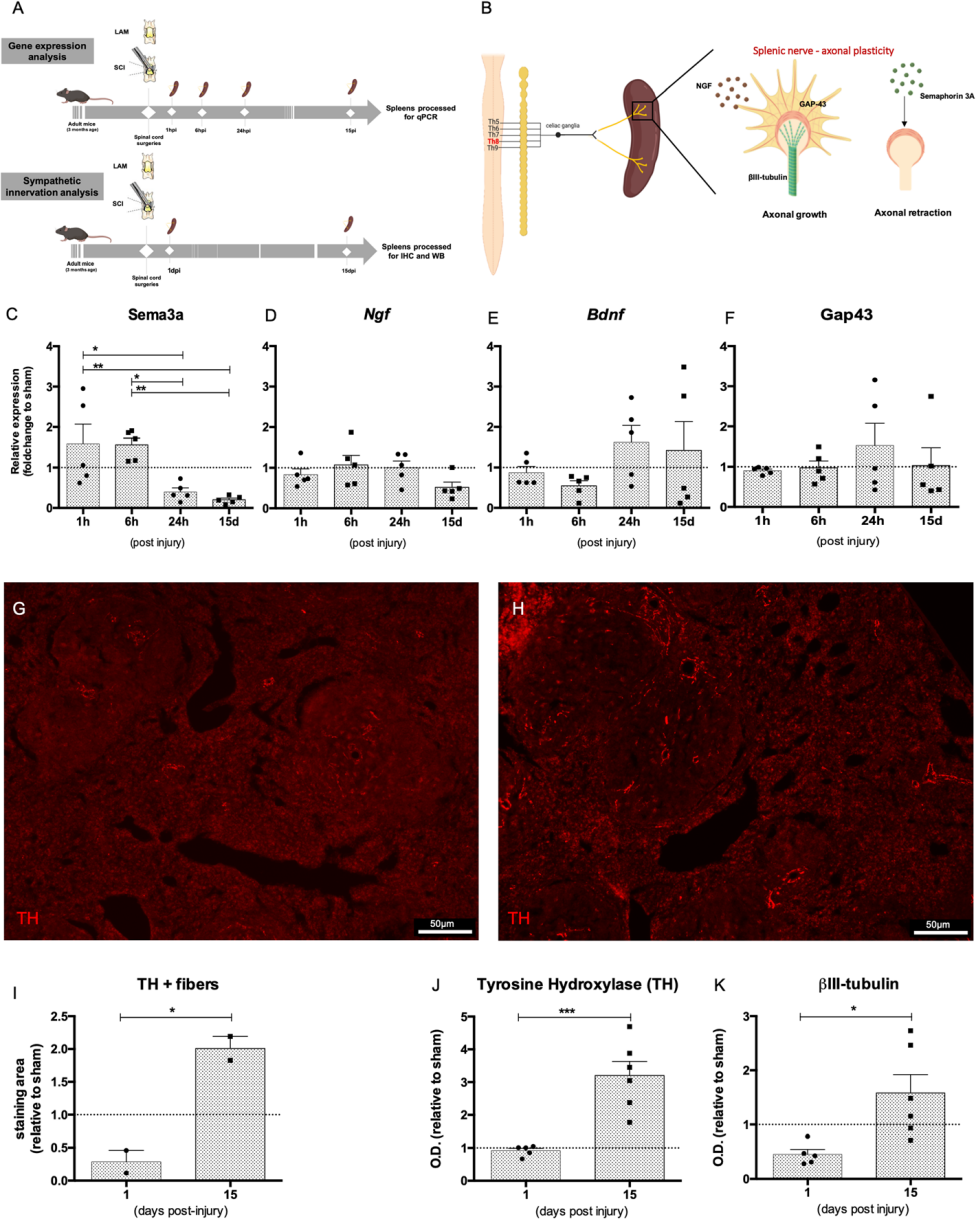
Mid-thoracic SCI alters gene expression and protein levels of molecules associated with axonal sprouting. **a** Experimental setup. **b** Schematic representation of the impact of a thoracic SCI in the sprouting of postganglionic neurons innervating the spleen. Created with BioRender.com. **c**–**f** Expression analysis of repulsive (**c**) and growth-associated (**d**–**f**) genes by qPCR in spleens harvested 1 h, 6 h, 24 h, and 15 days post-injury. Expression analysis was normalized to three housekeeping genes: *Gapdh*, *Hprt*, and *18s*, and variation to respective time-point sham control is shown. Results were pooled from two independent experiments. *N* (1 h) = 5; *n* (6 h) = 5; *n* (24 h) = 5; *n* (15 days) = 5. **g**–**i** Immunohistochemistry of TH+ positive fibers in the spleen at 1 (**g**) and 15 dpi (**h**). Scale 50 μm. **i** Area occupied by sympathetic fibers (TH+) in the spleen was quantified by positive TH immunostaining relative to respective time-point sham control. *N* (1 day) = 2; *n* (15 days) = 2. **j**, **k** Western blot analysis of TH (**j**) and βIII-tubulin (**k**) in the spleen harvested 1 and 15 days post-injury (dpi). Protein analysis was normalized for the housekeeping protein actin, and variation to respective time-point sham control is shown. Data representative of three independent experiments. *N* (1 day) = 5; *n* (15 days) = 6. Statistical tests: **c**–**f** one-way ANOVA; post-hoc Tukey’s multiple comparisons test. **i**, **j**, and **k** unpaired *t* test. Results expressed as mean ± SEM. *p < 0.05; **p < 0.01, and ***p < 0.001

Neuroplastic events after SCI involving preganglionic neurons have been documented [4] but less clear is whether and when similar morphological changes occur in postganglionic neurons innervating lymphoid organs. Although not being physically affected by SCI, postganglionic neurons can display morphological changes due to altered preganglionic signaling or by environmental molecules that signal back to the sympathetic nervous system [21]. Similarly to other organs, the spleen can become hyper-innervated weeks after SCI [14] in high-level SCI, but still unknown is what is the time-frame for splenic neuronal remodeling to occur and whether this phenomena also occurs after a mid-thoracic injury. We conducted a kinetic analysis for the first hours to weeks after SCI and tracked changes in the expression of environmental and axon-intrinsic signals associated with axonal growth or retraction in the spleen (Fig. 1a–k). We found that semaphorin 3A, a molecule known to induce retraction, (Fig. 1b) was progressively reduced in the spleen following SCI (*F*(4, 21) = 5.081; *p* = 0.005). Multiple comparison post hoc analysis showed that this reduction was most significant at 2 weeks after injury when comparing to the first and 6th hour after SCI with a *p* < 0.05 (Fig. 1c). We also did not detect significant changes in the expression levels of the neurotrophin nerve growth factor (NGF) and brain-derived neurotrophic factor (BDNF) (Fig. 1 d and e). The expression levels of growth-associated protein 43 (GAP-43), a protein present at the growth cone of growing/regenerating axons (Fig. 1b) was not significantly altered within this time-frame following injury (Fig. 1f). After measuring protein levels of βIII-tubulin and of tyrosine hydroxylase (TH), a protein associated with sympathetic fibers, we observed that both protein levels were significantly changed in the spleen after SCI (βIII-tubulin: *F*(2, 11) = 4.032; *p* = 0.04; TH: *F*(2, 11) = 13.06; *p* = 0.001) (Fig. 1 j and k; Fig. S1). Post hoc analyses showed that the levels of TH and βIII-tubulin were increased at 2 weeks when comparing to the levels of the first day (Fig. 1j and k; Fig. S1). Immunohistochemistry analysis comparing the density of TH+ fibers present in the parenchyma 15 days with those at 1 day post-injury (dpi), revealed changes between these time-points (*t*(df2) = 6.859; *p* = 0.021) (Fig. 1g–i). Collectively, these data demonstrate that a mid-thoracic compression injury leads to dynamic morphological changes in the spleen neuronal innervation. Although the genetic machinery may start early, these morphological changes are only evident weeks following SCI.

### Splenic NE levels are already raised 1 day after SCI

**Fig. 2:**
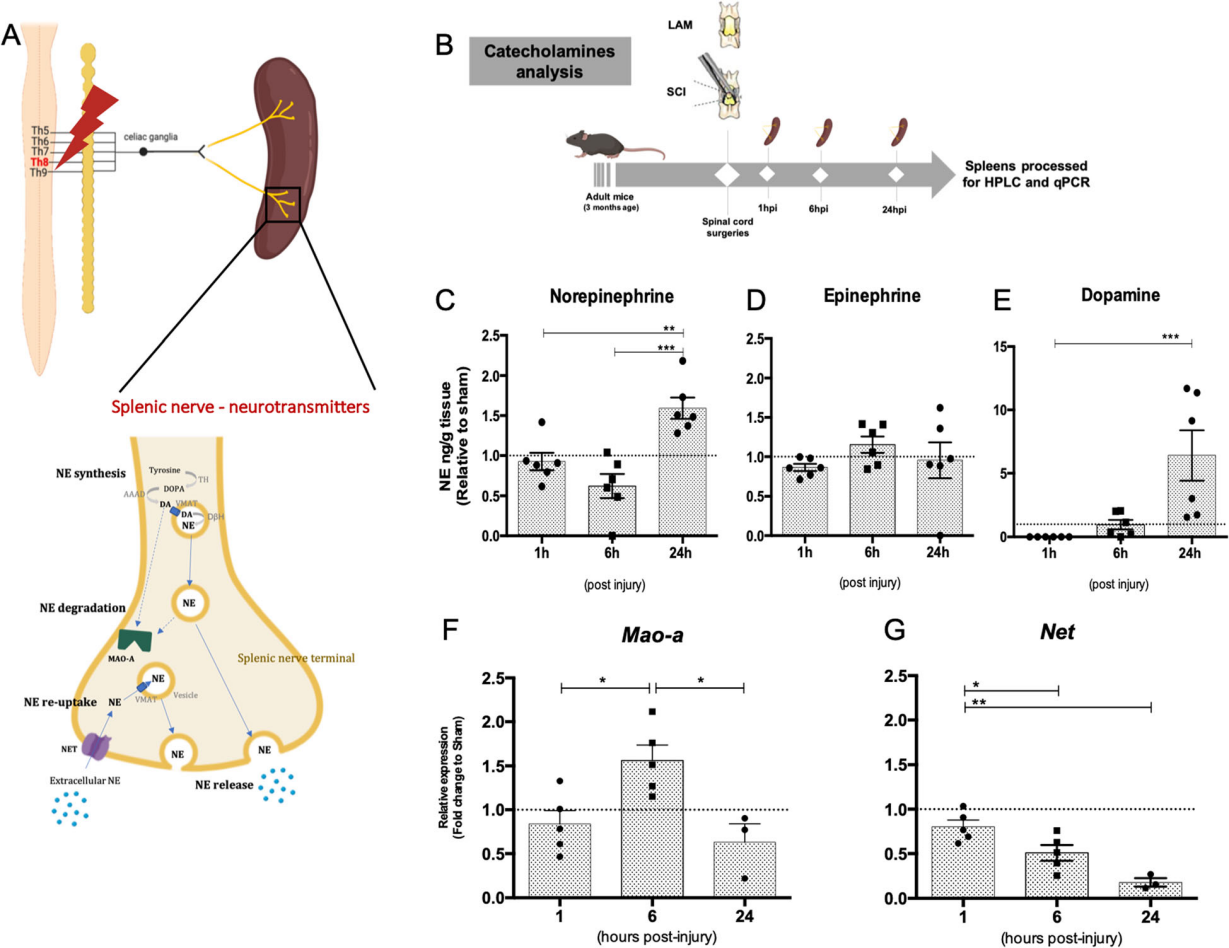
Thoracic SCI promotes an increase in splenic NE levels and alters the expression of NE metabolism-associated genes. **a** Schematic representation of the impact of a thoracic SCI in the neurotransmitters’ metabolism of postganglionic terminals in the spleen. Created with BioRender.com. **b** Experimental setup. **c**–**e** Catecholamine’s quantification in the spleen 24 hpi by high performance liquid chromatography (HPLC). Quantification data was normalized to spleen weight, and variation relative to respective time-point sham control is shown. Data pooled from two independent experiment. *N* (1 h) = 6; *n* (6 h) = 6; *n* (24 h) = 6. **f**, **g** Expression analysis of NE metabolism-associated genes by qPCR in spleens harvested 1, 6, and 24 hpi. Expression analysis was normalized to three housekeeping genes: *Gapdh*, *Hprt*, and *18s*, and variation relative to respective time-point sham control is shown. *N* (1 h) = 5; *n* (6 h) = 5; *n* (24 h) = 3. Statistical tests: **c**–**g** one-way ANOVA; post hoc Tukey’s multiple comparisons test. Results expressed as mean ± SEM. *p < 0.05; **p < 0.01; ***p < 0.001

Sympathetic terminals innervating the spleen are noradrenergic being NE the main secreted neurotransmitter at the spleen parenchyma (Fig. 2a). Previous studies have reported altered levels of NE in the spleen in response to SCI [2, 5, 13] weeks after the injury was performed. Here we show that increased levels of NE in the spleen can be detected as early as 1 dpi (*t* = 4.171; df = 6.134; *p* = 0.006) (Fig. 2c), with no changes in epinephrine (Fig. 2d). The levels of dopamine, the direct precursor for NE synthesis, were also significantly raised at 1 dpi (*t* = 2.741; df = 5.063; *p* = 0.040) (Fig. 2e). Looking for other correlates of local NE metabolism, we analyzed the expression levels of monoamine oxidase A (MAO-A), an enzyme responsible for NE degradation, and of norepinephrine transporter (NET), a NE-specific transporter, responsible for the re-uptake from the extracellular space by the pre-synaptic terminal. We found a significant variation in MAO-A expression levels during the first 24 h post-injury (hpi) (Fig. 2f). MAO-A expression levels are significantly increased at 6 hpi returning to basal levels at 24 h. NE re-uptake is also potentially affected 24 hpi as measurement of NET expression levels were progressively reduced being minimal at 24 hpi (Fig. 2g). Importantly, no changes in the spleen weight were found at this time-point (Fig. S2).

### Splenic neutrophils respond to sympathetic activation

**Fig. 3:**
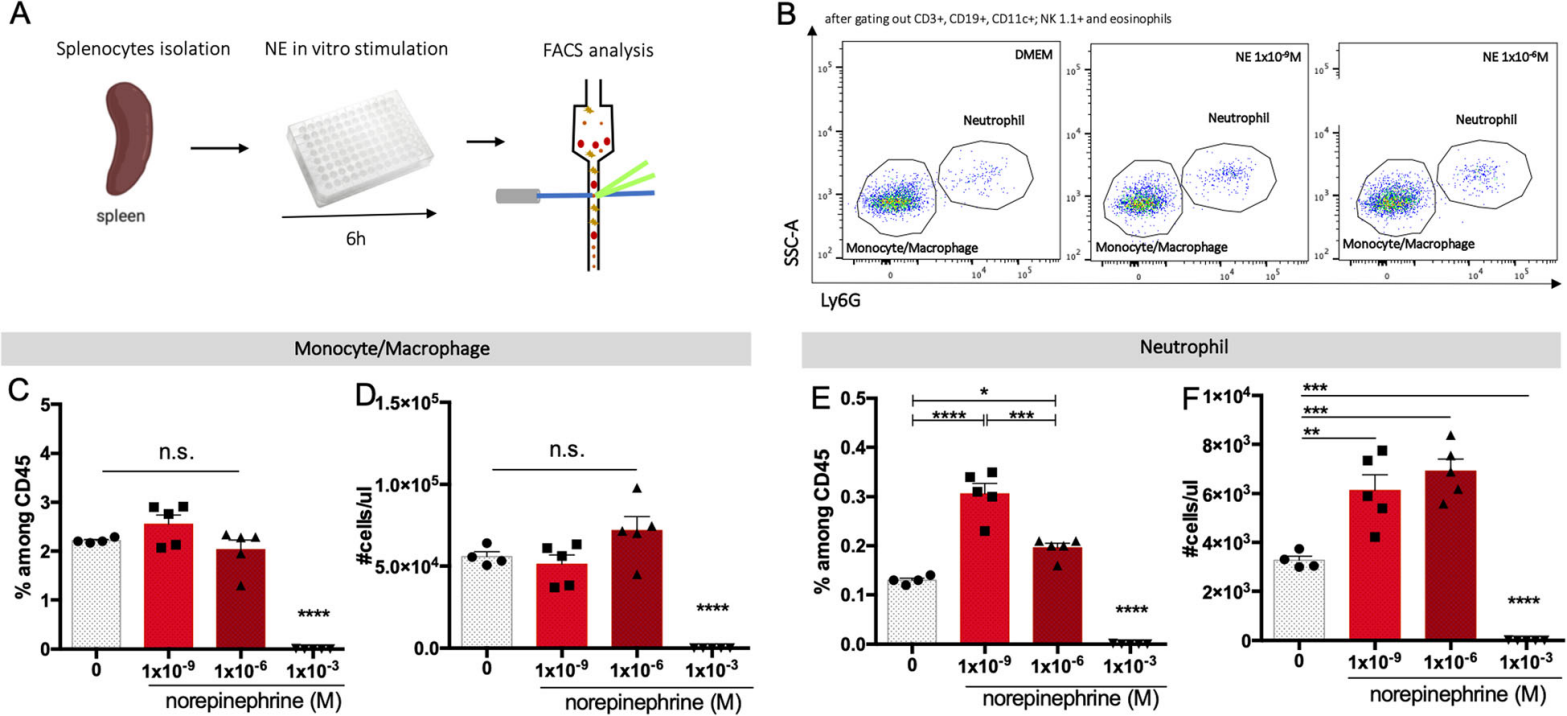
Lower doses of NE increase neutrophil population among splenocytes. **a** Schematic representation of in vitro assay and flow cytometry assessment. **b** Flow cytometry plots demonstrating monocytes/macrophages (SSC^low^Ly6G^−^) and neutrophil (SSC^high^Ly6G^+^)populations. **c**, **d** Frequency and concentration of neutrophils after NE stimulation. **e**, **f** Frequency and concentrations of monocytes/macrophages after NE stimulation. *N* (0 M) = 4; *n* (1 × 10^−9^) = 5; *n* (1 × 10^−6^) = 5; *n* (1 × 10^−3^) = 5. Data representative of three independent experiments. Statistical tests: **b**–**f** one-way ANOVA; post hoc Tukey’s multiple comparisons test. Results expressed as mean ± SEM. n.s. non-statistically significant; *p < 0.05; **p < 0.01; ***p < 0.001; ****p < 0.0001

Most of the studies demonstrating the impact of NE on immune cells focus mainly on lymphocytes [22]. Here, we assessed the impact of NE stimulation (Fig. 3a) in two major myeloid populations, monocytes/macrophage and neutrophils, since these are the first inflammatory cells infiltrating the spinal cord after injury both in humans [23] and rodents [24].

In line with previous studies demonstrating that NE leads to apoptosis of splenic immune cells [22], we observed that stimulation with higher doses (1 × 10^−3^ M) depletes both monocytes/macrophages (Fig. 3c and d) and neutrophils (Fig. 3e, f). Lower doses, however, had an opposite effect, specifically increasing the neutrophils’ population (Fig. 3b, e, and f). The frequency (F_(2, 11)_ = 36.57; *p* < 0.0001; post hoc: 0 vs 1 × 10^−9^ M—*p* < 0.0001; 0 vs 1 × 10^−6^ M—*p* < 0.05) and concentration (F_(2, 11)_ = 12.91; *p* = 0.0013; post hoc: (0 vs 1 × 10^−9^M/1 × 10^−6^M—*p* < 0.01) of neutrophils were significantly increased among splenocytes after lower-doses of NE stimulation (Fig. 3b, e, and f).

**Fig. 4:**
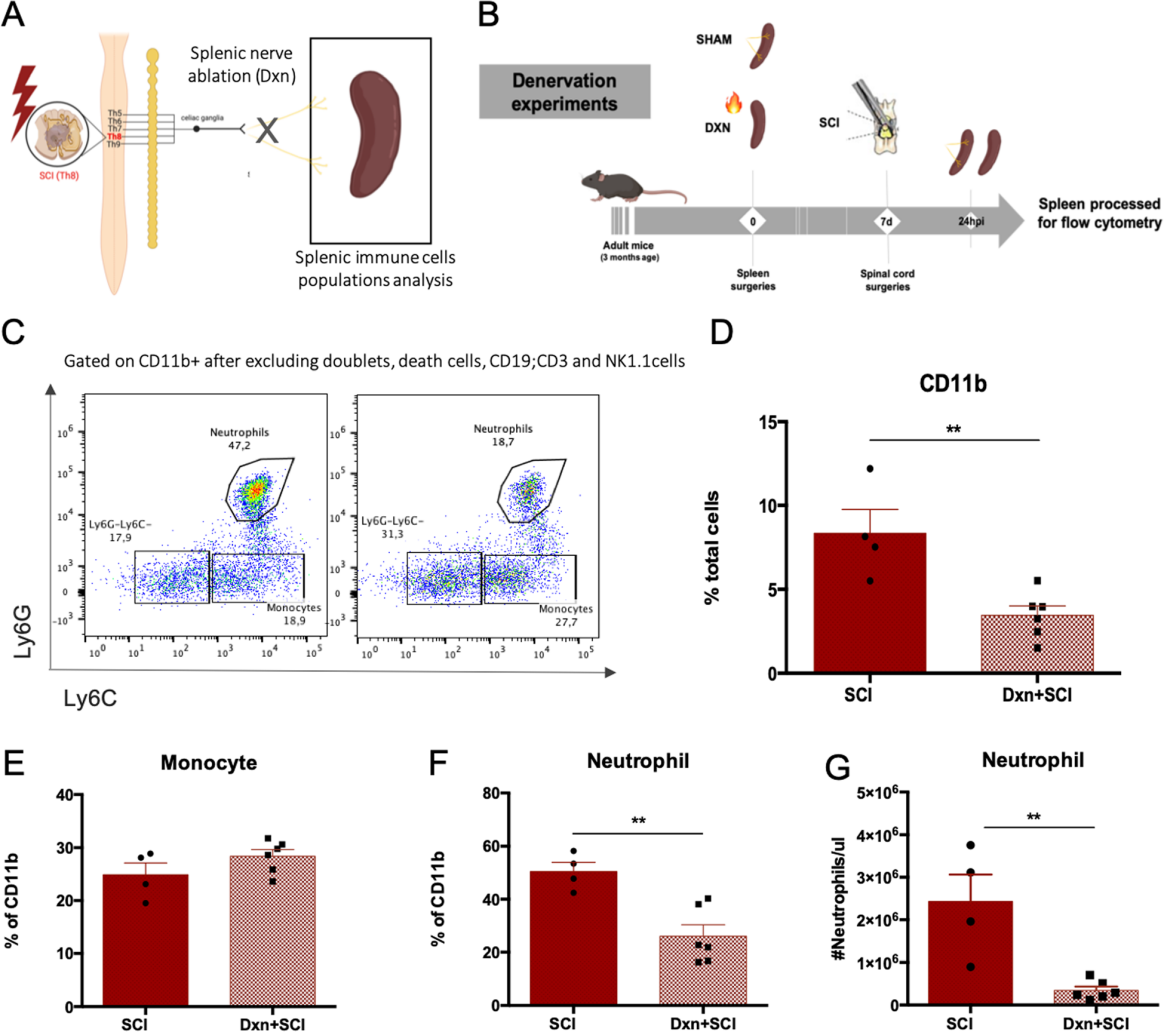
Less neutrophils in the spleen of denervated (Dxn) animals 24 hpi. **a** Schematic representation of the sympathetic signaling interruption by splenic denervation. Created with BioRender.com. **b** Experimental setup. **c** Flow cytometry plots demonstrating monocytes (CD11b^+^Ly6G^-^Ly6C^+^) and neutrophil (CD11b^+^Ly6G^+^Ly6C^+^) populations present in the spleen 24 hpi in SCI vs Dxn + SCI animals. **d**–**f** Frequency of myeloid (CD11b^+^) cells, monocytes (CD11b^+^Ly6G^-^Ly6C^+^), and neutrophils (CD11b^+^Ly6G^+^Ly6C^+^) present at the spleen 24 hpi in SCI and Dxn + SCI animals. **f** Concentration of neutrophils in the spleen 24 hpi in SCI vs Dxn + SCI animals. *N* (SCI) = 4; *n* (Dxn + SCI) = 6. Statistical tests: **d**–**g** unpaired *t* test. Results expressed as mean ± SEM. **p < 0.01

Then, we assessed whether the altered sympathetic signaling to the spleen 24 h after SCI had an effect to the splenic monocytes and neutrophil populations. We performed a selective sympathetic denervation of the spleen (Dxn), by surgical thermoablation of the splenic nerve [12] (Fig. S3A and B). Contrary to other sympathetic denervation approaches, such as the systemic administration of 6-OHDA or the resection of the splenic nerve, the thermoablation technique allows the possibility of isolating the specific contribution of the splenic sympathetic innervation while preserving other organs’ sympathetic terminals. Moreover, this procedure ablates the splenic nervous plexus without causing major alterations to the structure of the splenic artery, and the blood supply to the spleen is preserved. Even after 30 days of the splenic denervation, we observed a total ablation of sympathetic fibers in the spleen parenchyma (Fig. S3C-E) without causing major alterations to the spleen histoarchitecture (Fig. S3B) suggesting that no significant regeneration of these terminals occurs. Also, splenic denervation procedure did not affect the major lymphoid and myeloid populations of the spleen 7 days after the procedure (Fig. S4). Ablating the sympathetic signaling to the spleen (Fig. 4 a and b) led to less myeloid (CD11b+) cells present in the spleen after SCI when comparing to sham-operated animals (*t* = 3724; df = 8; *p* = 0.006) (Fig. 4d). Among CD11b^+^ cells, neutrophils but not monocytes were the most significantly altered populations by the absence of sympathetic signaling, with less cells being present at the spleen 24 hpi (% among CD45^+^: *t* = 4.052; df = 8; *p* = 0.0037); concentration: *t* = 4.075; df = 8; *p* = 0.0036) (Fig. 4c, e–g).

### Splenic sympathetic signaling is involved in the neutrophil infiltration of the spinal cord after injury

We next questioned whether these myeloid cell alterations observed in the spleen after SCI were relevant for the inflammatory cells infiltrating the spinal cord. The spleen harbors a pool of monocytes that play a central role in tissue injury [11], and in fact, the monocyte-derived macrophages found in the spinal cord parenchyma in the first week after SCI are originated from this splenic pool [17]. Although there are also several studies pointing for the existence of a splenic neutrophil pool important for fighting infections [9, 25–27], its putative role in tissue injury is still to be tested.

**Fig. 5:**
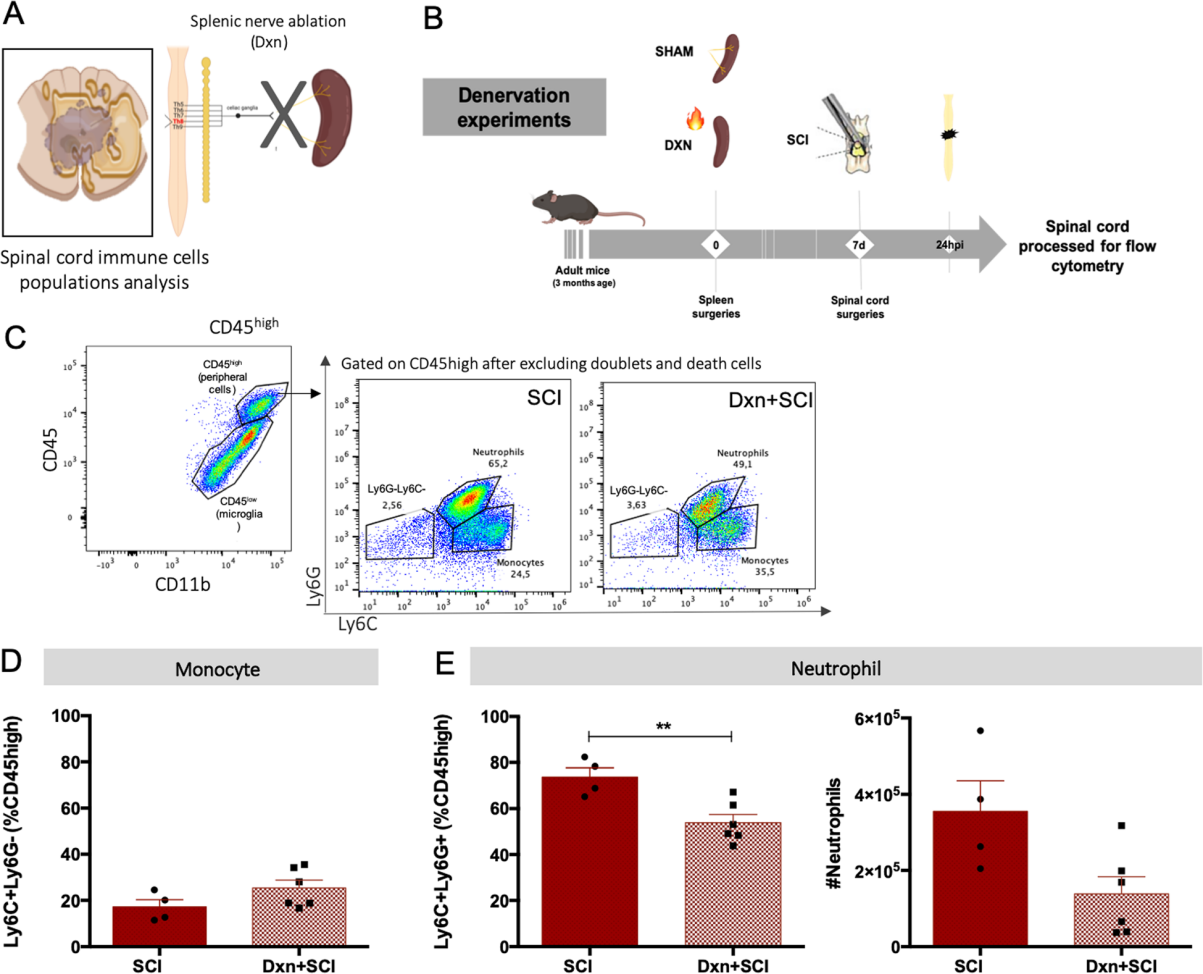
Splenic denervation led to less neutrophils present at the spinal cord 24 hpi. **a** Schematic representation of the sympathetic signaling interruption by splenic denervation. Created with BioRender.com. **b** Experimental setup. **c** Flow cytometry plots demonstrating gates defining CD45^high^ cells, monocytes (CD45^high^CD11b^+^Ly6G^-^Ly6C^+^), and neutrophil (CD45^high^CD11b^+^Ly6G^+^Ly6C^+^) populations present at the spinal cord 24 hpi in SCI vs Dxn + SCI animals. **d** Frequency of monocytes present at the spinal cord 24 hpi in SCI vs Dxn + SCI animals. **e** Frequency and concentration of neutrophils at the spinal cord 24 hpi in SCI vs Dxn + SCI animals. *N* (SCI) = 4; *n* (Dxn + SCI) = 6. Statistical tests: **d**, **e** unpaired *t* test. Results expressed as mean ± SEM. ***p < 0.001

For understanding if the spleen was relevant for intraspinal inflammation, we compared the inflammatory response mounted at the spinal cord 24 hpi of splenectomized to that of sham-operated animals (Fig. S5). We observed that the frequencies of neutrophils at the spinal cord of splenectomized animals were sharply reduced (Fig. S5B). Using again the splenic sympathetic denervation model, we tested whether the sympathetic signaling was involved in the neutrophil infiltration of the injured spinal cord (Fig. 5 a and b; Fig. S6). In line with previous studies [23, 24], we observed that at 24 hpi the majority of CD45^high^ positive cells (infiltrating inflammatory cells and residual activated microglia) were neutrophils (Fig. 5c). Although neutrophils were still present at the spinal cord 24 h after the injury, the percentage was significantly lower in denervated animals when comparing to sham-operated animals (*t* = 3.; df = 10; *p* = 0.0035) (Fig. 5c–e).

## Discussion

The intraspinal inflammatory response mounted after SCI lacks many features of a controlled wound healing response, being exaggerated in its magnitude and prolonged and non-resolving in its duration. Several systemic immune alterations have been documented after SCI. Studies demonstrate that SCI can promote pro-inflammatory responses damaging peripheral organs [28, 29], to others showing deficient immune responses to pathogens or severe immunosuppression [1, 3–5, 13]. However, the involvement of a systemic immune dysregulation into the dysfunctional aspects of the intraspinal inflammatory response has been unappreciated being mainly attributed to injury environmental factors.

In this study, we identify the involvement of a heightened sympathetic nervous system functioning that was previously associated with some alterations of the immune system function [2, 3, 5, 22], with the frequencies of neutrophils present at the spinal cord after injury.

### SCI promotes a heightened signaling within the spinal-to-spleen sympathetic circuitry

SCI increases sympathetic tone to peripheral organs perturbing their correct functioning. Lymphoid organs are not an exemption, and altered levels of NE were detected in the spleen weeks after the injury [2, 13]. Our work demonstrates that variations in NE levels can already be detected at the spleen in the first 24 hpi. Interestingly, we found also variations in the expression of enzymes and transporters associated with NE degradation and re-uptake suggesting local regulation of NE levels. For instance, at 6 hpi, MAO-A expression peaks returning to initial levels at 24 h. In support of local degradation of NE, the levels of this neurotransmitter are lower also at this time-point. NET expression is progressively reduced in line with the increased levels of NE, which could be a reflection of less re-uptake of this neurotransmitter leading to more extracellular availability. The increased levels of dopamine detected at this time-point could also suggest de novo biosynthesis since this neurotransmitter is the direct precursor of NE. However, immune cells could also be the source of dopamine since they are also capable of producing this neurotransmitter with an impact in their function [30], so a non-neuronal source cannot be discarded.

Morphological changes in the sympathetic innervation of the spleen, however, were not found at 24 hpi. In line with previous reports [14], we observed a progressive sprouting of the splenic sympathetic innervation that could be detected at 15 dpi. Importantly, here we report variation in the expression levels of guidance cues in the spleen that could drive splenic innervation remodeling. Surprisingly, we did not observe variations in the expression levels of NGF, a neurotrophic factor known to induce growth in peripheral neurons, but found a progressively reduced expression of semaphorin-3A, a chemorepellent cue for axons. We observed downregulation of this cue as early as 24 hpi but was maximal at 15 dpi, the same time-point where we observed increased density of sympathetic terminals in the spleen parenchyma. Semaphorin 3A has also immunomodulatory functions by downregulating immune responses and can be produced by immune cells [31, 32] although this molecule is mostly known for its axonal guidance role, particularly during neurodevelopment and regeneration. This dual role is particularly interesting in the context of neuroimmune interactions since the reduction of this molecule could be both associated with increased sprouting of the splenic innervation or to an enhancement of local immune responses. Unexpectedly, we did not find any major alterations in gene expression of the axon-intrinsic molecule Gap-43 associated with the growth-cone. However, our analysis focused in the mRNA changes located at the spleen and did not consider the mRNA levels at the postganglionic neuron soma which is located at the celiac ganglia. Although *Gap43* mRNA can be found at axon terminals for local protein synthesis [33], we cannot exclude that the quantities may be low for detecting significant variations.

Our temporal characterization of the changes in the splenic innervation following injury shows that there are environmental cues that may contribute for the splenic sympathetic sprouting after a mid-thoracic SCI. However, although the gene machinery of signals driving axonal sprouting may start early at 24 hpi, most of the morphological changes are observed at latter time-points.

### The splenic sympathetic signaling is involved in acute neutrophil infiltration after SCI

Monocyte-derived macrophages and neutrophils are key for the acute intraspinal inflammatory response after SCI. Although the spleen has been identified as the source of the first monocytes infiltrating the spinal cord [17], neutrophils are generally assumed to be originated in the bone-marrow and blood circulation. In contrast with this assumption, our data demonstrates that splenectomy prior to SCI induces a sharp reduction in the percentage of neutrophils present at the spinal cord after injury, suggesting the involvement of the spleen in SCI-associated neutrophil infiltration. The presence of progenitors, immature and mature neutrophils in the spleen, and their functional relevance both in physiological and pathological contexts have been consistently demonstrated [25–27, 34]. The spleen is one of the organs exhibiting more neutrophil infiltration at steady-state exhibiting a non-random extravascular distribution [35]. These extravascular neutrophils in the spleen are either located at the marginal zone where they help B cell stimulation and antibody-independent of T cell production [27], or in the red pulp [26, 35] where they were shown to be important for helping macrophages in the phagocytosis of bacteria [26]. In the red pulp, two morphological and functionally distinct neutrophil populations were observed in the spleen, a static and immature population expressing Ly6G^int^, and other expressing Ly6G^high^—a mobile population that actively scans the spleen environment [26].

Sympathetic nerves and catecholamines are known to modulate immune responses [9, 10, 36–42] orchestrating leukocyte recruitment to tissues [7, 8], myelopoiesis [9], and neutrophil chemotaxis and phagocytosis [36]. For dissecting the specific involvement of the splenic sympathetic signaling to the SCI-immune alterations, we thermoablated the splenic nerve prior to SCI and tracked monocytes and neutrophils in the spleen and spinal cord 24 hpi. We observed a reduction in neutrophil infiltration after SCI in the absence of splenic sympathetic signaling being the first demonstration that the sympathetic signaling is, at least partially, involved in the numbers of peripheral inflammatory cells infiltrating the spinal cord. Also, we observed less neutrophils in the spleen 24 hpi in animals with splenic nerve ablation. One possibility would be that the lack of NE prevents the egress of hematopoietic stem cells from the bone marrow to the spleen, as NE from sympathetic terminals was shown to be critically involved in this phenomena [10]. However, when we stimulated splenocytes in vitro with NE, we observed a significant increase in the number of neutrophils, suggesting that rather than recruiting stem cells, NE is able to trigger local proliferation/maturation giving rise to more neutrophils from local cellular populations. Still to understand is whether the increased number of neutrophils are a reflection of extramedullary myelopoiesis or from more maturation of immature neutrophils present at the spleen [25, 26]. The spleen can rapidly provide a pool of mature effector neutrophils in cases of infection through local proliferation/maturation. In response to sepsis, it was shown that although the bone marrow exhibited a 2- to 4-fold increase in immature neutrophils, the spleen presented a 10-fold increase [34], while other study demonstrated that in response to *Streptococcus pneumoniae*, splenic immature neutrophils rapidly proliferate replenishing the mature neutrophil pool [26]. Still to understand is if SCI can also trigger splenic local proliferation/maturation of neutrophils.

Another hypothesis is that the spleen may serve as a site for terminal imprinting of neutrophils shaping their effector functions. In line, neutrophils isolated from splenectomized patients presented less chemotaxis and phagocytic capacity in vitro [43]. Also in the spleen, it was reported that neutrophils can differentiate into myeloid-derived suppressor cells contributing for cancer progression [44]. After SCI, many cytokines and chemokines important for activating and modulating inflammation are increased at the spleen [13], and neutrophils isolated from SCI patients exhibit alterations in their activity such as more oxidative activity [29].

Curiously, we found that neutrophils may respond differently to varying NE concentrations. While neutrophils were increased after 10^−9^ M and 10^−6^ M NE stimulation, their number starkly dropped after NE 10^−3^ M. These data support previous studies showing that the effects of NE on myeloid cells vary according to the concentration. One example is the effect of even lower doses of NE on monocytes. While low doses enhanced migratory capacity [40], higher doses prevented the migration [42]. Another example is the effect of short stimulation of NE being able to polarize neutrophils to a N2 phenotype exhibiting a reduction on the expression of genes associated with cytoskeleton remodeling and inflammation. Conversely, prolonged stimulation triggered a pro-inflammatory profile in neutrophils [36]. The differential effect of NE on myeloid cells according to concentration or time of exposure is relevant in the context of SCI where the varying levels of NE throughout time might have a differential impact in neutrophils immune responses.

As with the majority of the SCI research, this work only uses females, being a limitation for the generalization of the results. The spontaneous neurological recovery from CNS trauma, and specifically from SCI is enhanced in females than in male mice [45] although the exact mechanism is still unknown. Also, the immune system displays sexual dimorphism, and differences in the genes expressed by immune cells from males or females have been described [46].

## Conclusion

In summary, we observed temporal changes in the sympathetic signaling to the spleen after a mid-thoracic SCI. Furthermore, changes in the sympathetic signaling conveyed by the splenic nerve are associated with the acute neutrophil infiltration of the injured spinal cord. These findings should pave the way for future research aiming at exploring the spleen as a peripheral target for intraspinal immunomodulation.

## Supplementary information

**Additional file 1: Fig. S1.** Western blots for tyrosine hydroxylase and βIII-tubulin. Protein of interest optical density (O.D.) was normalized for the actin O.D.

**Additional file 2: Fig. S2.** Spleen weight and cell concentration remain unaltered at 24 h after SCI. A – Spleen weight is similar between control, laminectomy (sham-operated) and SCI animals at 24 hpi and also after normalizing for body weight (B). C - Concentration of splenocytes between laminectomy and SCI remained the same at 24 hpi. Results expressed as mean +/- S.E.M.

**Additional file 3: Fig S3.** Thermoablation of the splenic nerve does not lead to long-term major gross alterations in spleen and ablates all sympathetic terminals innervating the spleen parenchyma. A and B – The splenic artery (containing the splenic nerve) was dissected after de denervation procedure and prepared for whole-mount immunostaining (TH) for confirmation of the denervation site. The denervation site was located right before the first bifurcation of the splenic artery. Scale bar - 200 μm. C-E – In another set of animals, spleens were harvested 30 days after splenic denervation and immunostained for TH for excluding any long-term re-innervation phenomena. No sympathetic fibers could be observed 30 days after Dxn. Scale bar - 100 μm. F - Hematoxylin-eosin staining of spleens from denervated or sham-operated mice 30 days after Dxn. No major alterations in the histoarchitecture of the spleen can be observed 30d after denervation. Results expressed as mean +/- S.E.M.

**Additional file 4: Fig S4.** Lymphoid and myeloid populations of the spleen remain unaltered 7 days after the splenic denervation procedure. The frequency of the main lymphoid (T and B lymphocytes) and myeloid populations (monocytes (CD11b+Ly6G-Ly6C+) and neutrophils (CD11b+Ly6G+Ly6C+)) were analyzed 7d after the thermoablation of the splenic innervation or sham surgery. A - Experimental setup. B – Frequency of T lymphocytes (CD3+ cells). C – Frequency of B lymphocytes (CD19+). D- Frequency of myeloid cells (CD11b+). E – Frequency of neutrophils (CD11b+Ly6G+Ly6C+). F – Frequency of monocytes (CD11B+Ly6G-Ly6C+). N (sham) = 5; n (Dxn) = 8. Statistical tests: B-F – unpaired t-test. Results expressed as mean +/- S.E.M.

**Additional file 5: Fig S5.** Splenectomy prevents neutrophil’s infiltration 24 h after SCI. A– Schematic representation of the sympathetic signaling interruption by splenectomy. The infiltration of inflammatory cells after SCI was analyzed 24 h after SCI in mice with and without the spleen (sham surgery vs splenectomy) and compared to laminectomy and control animals. Created with BioRender.com. B – Single-cell suspensions of the spinal cord were analyzed by flow cytometry for the main infiltrative innate myeloid cells through the expression of CD11b^+^CD45^high^ (infiltrative innate myeloid cells); Ly6G^+^Ly6C^+^ (neutrophils) and Ly6G^-^Ly6C^+^ (both gated on CD11b^+^CD45^+^) monocytes. N (Ctrl)= 3; n (lam) =3; n (SCI) = 2; n (SCI+Splx) = 4. Statistical tests: B – two-way ANOVA with Sidak’s multiple comparisons test. Results expressed as mean +/- S.E.M. n.s – non-significant. * p<0.05; ** p<0.01. Mean values expressed as S.E.M.

**Additional file 6: Fig S6.** Gating strategy used for flow cytometry analysis of spinal cord single-cell suspensions. Doublets were excluded by FSC-A vs FSC-H scatter. Live leukocytes were gated after excluding 7-AAD+ cells and selecting CD45^+^ cells. Infiltrative leukocytes (and possibly some activated microglia), were selected by high expression of CD45 and CD11b. Neutrophils were selected by double-positive expression of Ly6G and Ly6C.

## Data Availability

The datasets used and/or analyzed during this study are available on reasonable request from the corresponding author.

## Abbreviations

SCI: Spinal cord injury
NE: Norepinephrine
IML: Intermediolateral nucleus
HPA: Hypothalamic pituitary nucleus
PBS: Phosphate-buffered saline
PFA: Paraformaldehyde
OCT: Optimal cutting temperature
MIA: Multi-image alignment
DMEM: Dulbecco’s modified eagle medium
FCS: Fetal calf serum
ACK: Ammonium-chloride potassium
FACS: Fluorescence-activated cell sorting
BSA: Bovine serum albumin
TH: Tyrosine hydroxylase
DAPI: 4′,6-Diamidino-2-phenylindole
HPLC: High performance liquid chromatography
RPM: Rotations per minute
qPCR: Real-time polymerase chain reaction
RNA: Ribonucleic acid
HPRT: Hypoxanthine-guanine phosphoribosyltransferase
GADPH: Glyceraldehyde 3-phosphate dehydrogenase
SEM: Standard error of the mean
NGF: Nerve growth factor
BDNF: Brain-derived neurotrophic factor
GAP-43: Growth-associated protein 43
DPI: Days post-injury
HPI: Hours post-injury
MAO-A: Monoamine oxidase A
NET: Norepinephrine transporter
DXN: Denervation
