# Supplementary figures and images for: Splenic sympathetic signaling contributes to acute neutrophil infiltration of the injured spinal cord
Source: J Neuroinflammation. 2020 Sep 23;17:282. doi: 10.1186/s12974-020-01945-8 (PMC7513542; doi:10.1186/s12974-020-01945-8)

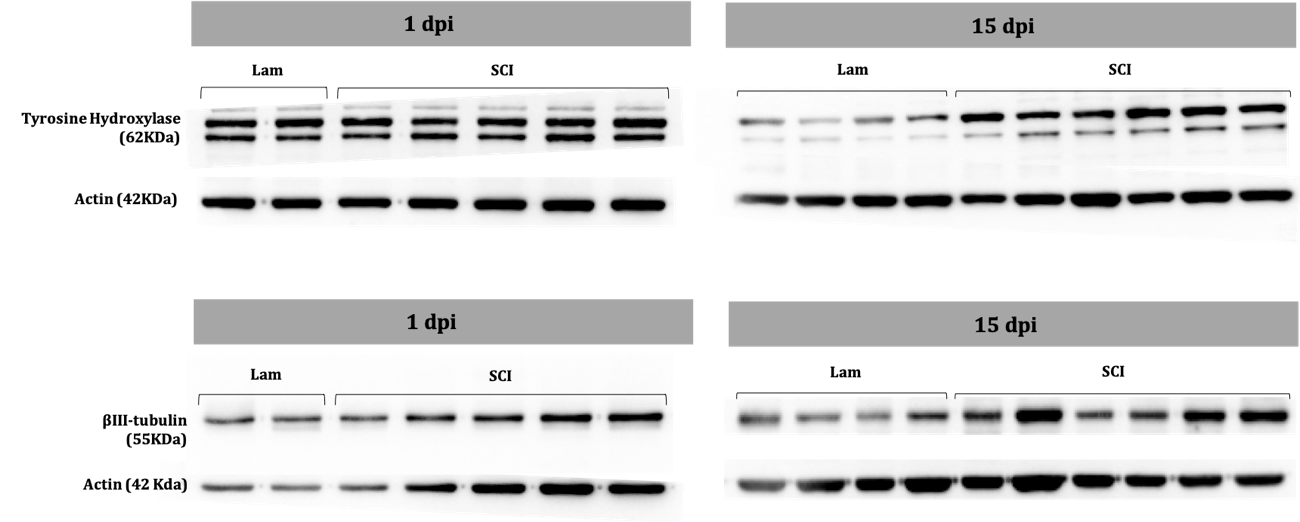

Supplement: Supplementary file 1 — Additional file 1: Fig. S1. Western blots for tyrosine hydroxylase and βIII-tubulin. Protein of interest optical density (O.D.) was normalized for the actin O.D. [file 12974_2020_1945_MOESM1_ESM.docx]

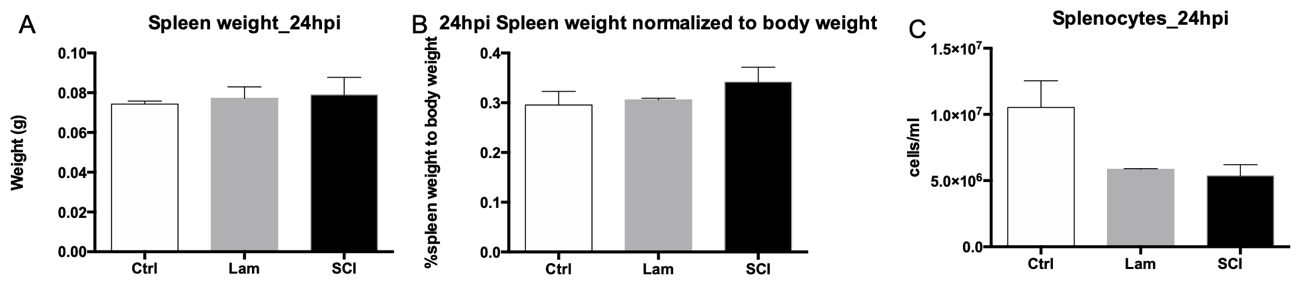

Supplement: Supplementary file 2 — Additional file 2: Fig. S2. Spleen weight and cell concentration remain unaltered at 24 h after SCI. A – Spleen weight is similar between control, laminectomy (sham-operated) and SCI animals at 24 hpi and also after normalizing for body weight (B). C - Concentration of splenocytes between laminectomy and SCI remained the same at 24 hpi. Results expressed as mean +/- S.E.M. [file 12974_2020_1945_MOESM2_ESM.docx]

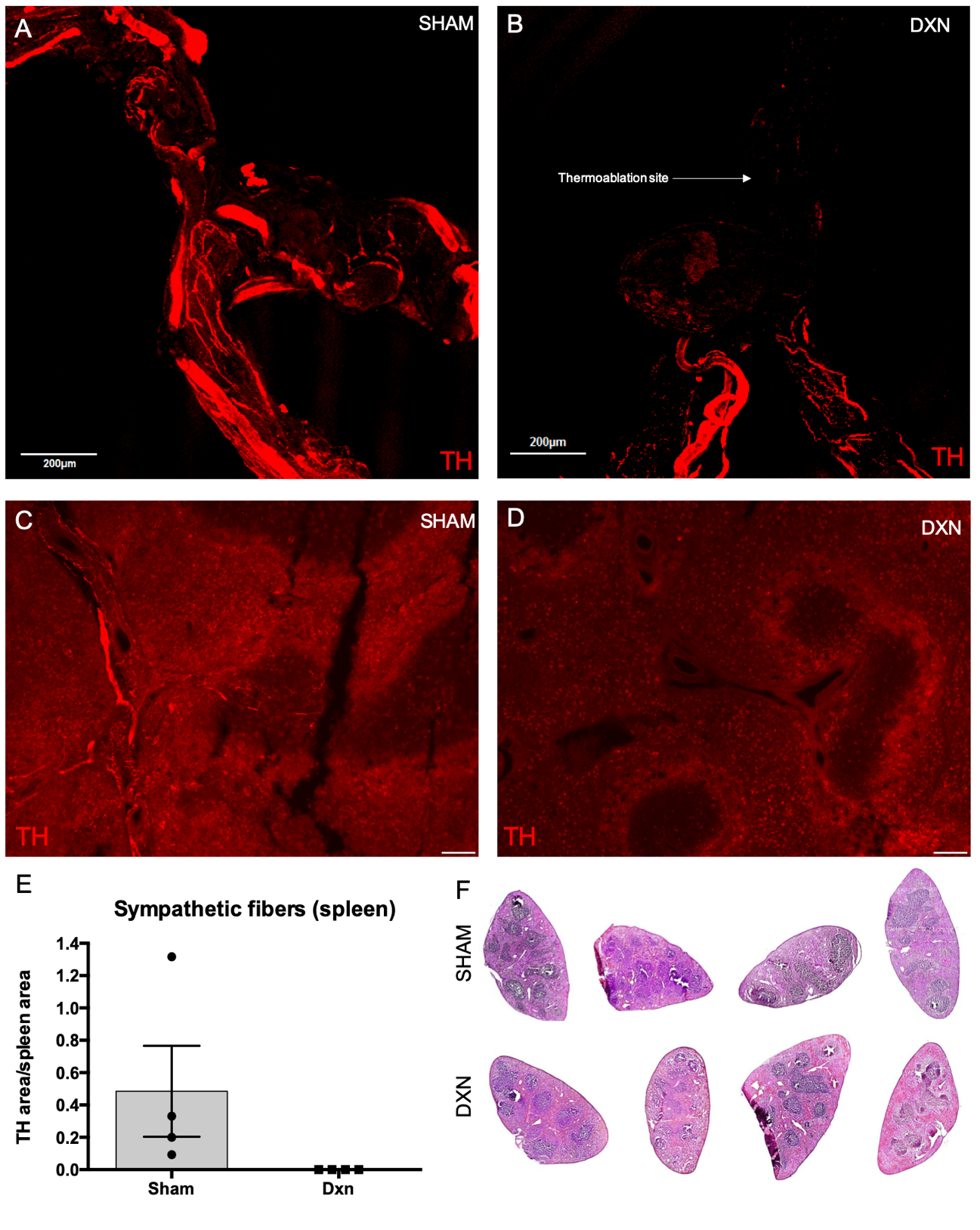

Supplement: Supplementary file 3 — Additional file 3: Fig S3. Thermoablation of the splenic nerve does not lead to long-term major gross alterations in spleen and ablates all sympathetic terminals innervating the spleen parenchyma. A and B – The splenic artery (containing the splenic nerve) was dissected after de denervation procedure and prepared for whole-mount immunostaining (TH) for confirmation of the denervation site. The denervation site was located right before the first bifurcation of the splenic artery. Scale bar - 200 μm. C-E – In another set of animals, spleens were harvested 30 days after splenic denervation and immunostained for TH for excluding any long-term re-innervation phenomena. No sympathetic fibers could be observed 30 days after Dxn. Scale bar - 100 μm. F - Hematoxylin-eosin staining of spleens from denervated or sham-operated mice 30 days after Dxn. No major alterations in the histoarchitecture of the spleen can be observed 30d after denervation. Results expressed as mean +/- S.E.M. [file 12974_2020_1945_MOESM3_ESM.docx]

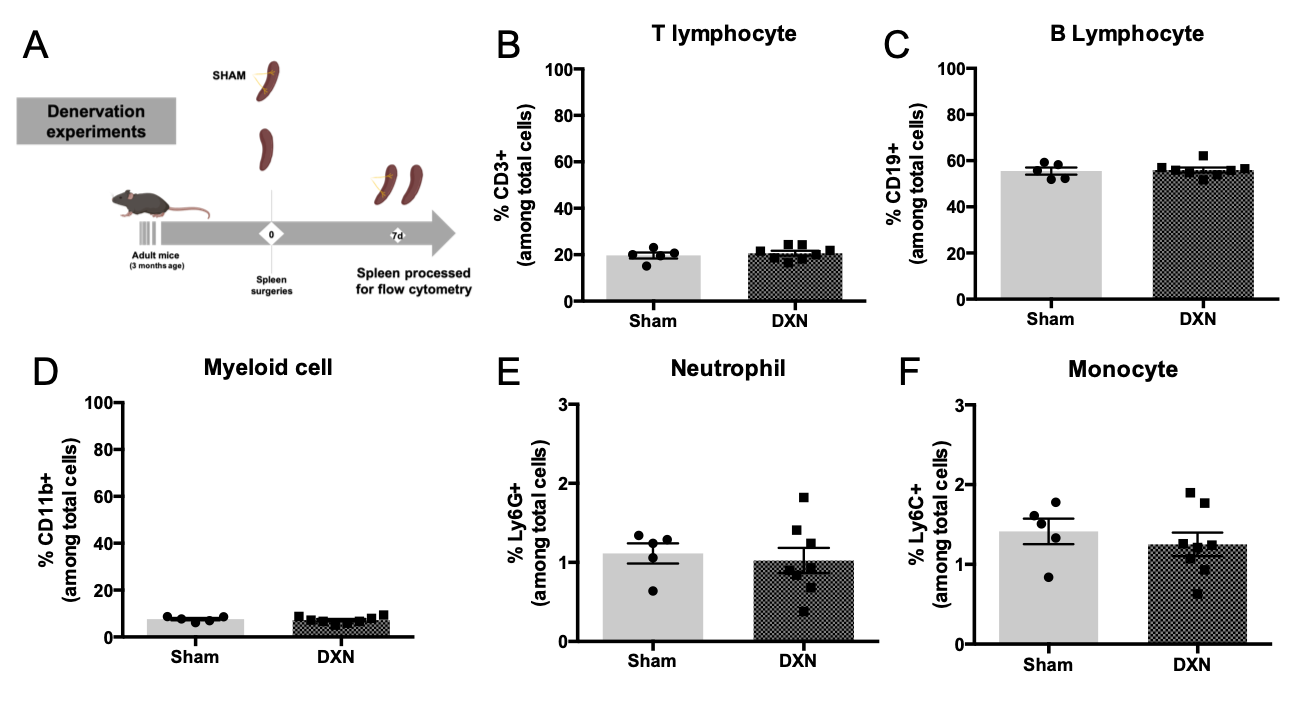

Supplement: Supplementary file 4 — Additional file 4: Fig S4. Lymphoid and myeloid populations of the spleen remain unaltered 7 days after the splenic denervation procedure. The frequency of the main lymphoid (T and B lymphocytes) and myeloid populations (monocytes (CD11b+Ly6G-Ly6C+) and neutrophils (CD11b+Ly6G+Ly6C+)) were analyzed 7d after the thermoablation of the splenic innervation or sham surgery. A - Experimental setup. B – Frequency of T lymphocytes (CD3+ cells). C – Frequency of B lymphocytes (CD19+). D- Frequency of myeloid cells (CD11b+). E – Frequency of neutrophils (CD11b+Ly6G+Ly6C+). F – Frequency of monocytes (CD11B+Ly6G-Ly6C+). N (sham) = 5; n (Dxn) = 8. Statistical tests: B-F – unpaired t-test. Results expressed as mean +/- S.E.M. [file 12974_2020_1945_MOESM4_ESM.docx]

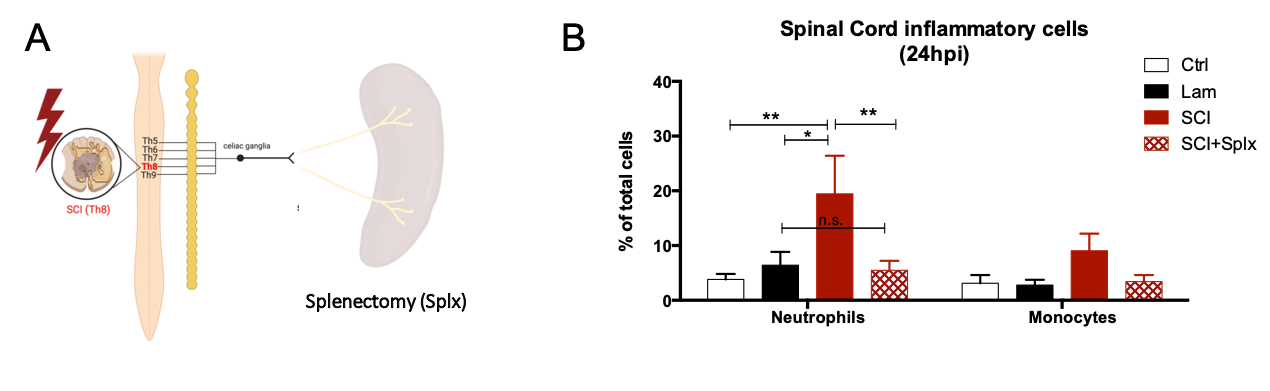

Supplement: Supplementary file 5 — Additional file 5: Fig S5. Splenectomy prevents neutrophil’s infiltration 24 h after SCI. A– Schematic representation of the sympathetic signaling interruption by splenectomy. The infiltration of inflammatory cells after SCI was analyzed 24 h after SCI in mice with and without the spleen (sham surgery vs splenectomy) and compared to laminectomy and control animals. Created with BioRender.com. B – Single-cell suspensions of the spinal cord were analyzed by flow cytometry for the main infiltrative innate myeloid cells through the expression of CD11b+CD45high (infiltrative innate myeloid cells); Ly6G+Ly6C+ (neutrophils) and Ly6G-Ly6C+ (both gated on CD11b+CD45+) monocytes. N (Ctrl)= 3; n (lam) =3; n (SCI) = 2; n (SCI+Splx) = 4. Statistical tests: B – two-way ANOVA with Sidak’s multiple comparisons test. Results expressed as mean +/- S.E.M. n.s – non-significant. * p<0.05; ** p<0.01. Mean values expressed as S.E.M. [file 12974_2020_1945_MOESM5_ESM.docx]

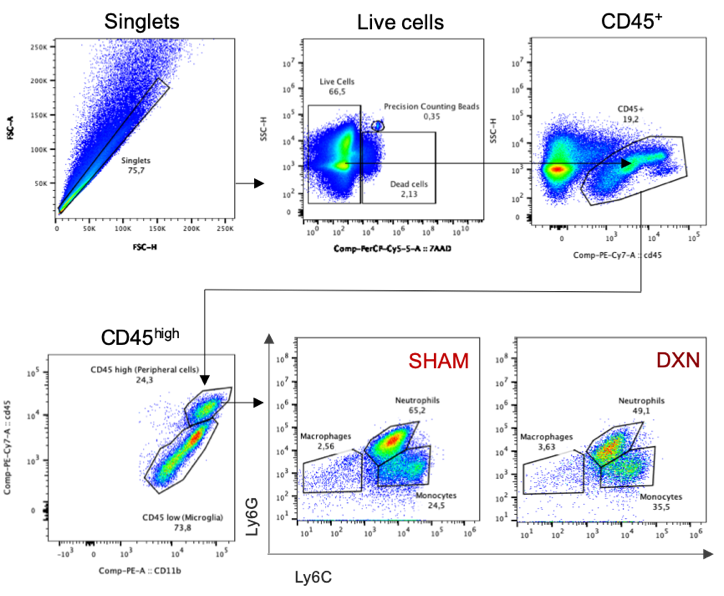

Supplement: Supplementary file 6 — Additional file 6: Fig S6. Gating strategy used for flow cytometry analysis of spinal cord single-cell suspensions. Doublets were excluded by FSC-A vs FSC-H scatter. Live leukocytes were gated after excluding 7-AAD+ cells and selecting CD45+ cells. Infiltrative leukocytes (and possibly some activated microglia), were selected by high expression of CD45 and CD11b. Neutrophils were selected by double-positive expression of Ly6G and Ly6C. [file 12974_2020_1945_MOESM6_ESM.docx]
